# Enhancing proton therapy quality assurance with custom‐designed Octopoint phantom and Gafchromic film

**DOI:** 10.1002/acm2.70156

**Published:** 2025-07-14

**Authors:** Xueyan Tang, Chris J. Beltran, Keith M. Furutani, Graham S. Gilson, Jon Gustafson, Michael G. Herman, Shima Ito, Jed E. Johnson, Jon J. Kruse, Kenneth M. Long, Daniel W. Mundy, Nicholas B. Remmes, Ali M. Tasson, Thomas J. Whitaker, Witold Matysiak, Erik J. Tryggestad

**Affiliations:** ^1^ Department of Radiation Oncology Mayo Clinic Rochester Minnesota USA

**Keywords:** Isocentricity, quality assurance, spot scanning proton therapy

## Abstract

**Background:**

Proton therapy offers precise tumor targeting while minimizing damage to surrounding healthy tissue, making it especially valuable for treating tumors near critical organs and in pediatric patients. However, its success depends on accurate beam delivery, requiring rigorous quality assurance (QA) to maintain treatment precision and effectiveness.

**Purpose:**

This study aims to standardize the measurement of key aspects of proton therapy delivery, such as gantry and couch isocentricity, spot position accuracy, and spot size consistency. The goal is to enhance the delivery accuracy of proton therapy across various clinical settings, improving patient outcomes.

**Methods:**

The QA framework uses the Octopoint phantom, Gafchromic film, and spot position monitor (SPM) log data to evaluate proton beam isocentricity, spot position, and spot size. The Octopoint phantom, made from acrylic, was used with Gafchromic films at various gantry and couch angles to measure isocentricity. MATLAB tools were used to analyze spot positions, and SPM logs provided verification. Sensitivity tests were conducted to assess the system's response to intentional shifts and errors, ensuring alignment with clinical QA standards.

**Results:**

The Octopoint phantom's stepwise two‐dimensional (2D) fitting process, validated against film‐measured dose profiles, accurately identified beam and ball bearing (BB) centroid locations. Isocentricity tests conducted over 8 months across four gantries demonstrated consistent beam‐to‐BB radial offsets. The phantom showed excellent repeatability, with a maximum standard deviation of 0.1 mm across various couch‐gantry combinations. Sensitivity testing across all axes revealed a strong alignment between intended shifts and measured values. Over the course of a year, film measurements tracked spot position and size consistency, with deviations remaining within acceptable clinical limits. Comparisons with SPM data further confirmed the system's reliability in maintaining beam accuracy across different gantry angles and energy levels.

**Conclusions:**

This study presents a reliable QA framework for ensuring precision in proton therapy delivery. By combining the Octopoint phantom, Gafchromic film analysis, and SPM log file evaluation, we effectively measured isocentricity, spot position accuracy, and spot size stability. The framework demonstrated adaptability across various clinical QA tasks, enhancing the accuracy and safety of proton therapy treatments.

## 1 INTRODUCTION

Using proton pencil beams in radiation therapy provides a unique ability to sculpt dose in three dimensions, given their small size and defined range.^1^ This precision stems from the unique properties of protons, which deposit the majority of their energy at a specific depth, known as the Bragg peak.^2^ This allows for highly localized treatment, making proton therapy especially valuable for tumors near critical organs such as the brain, spine, or heart, where preserving healthy tissue is crucial.^3^ Additionally, proton therapy is beneficial for pediatric patients, who are more vulnerable to the long‐term effects of radiation.^4^ By reducing unnecessary radiation exposure, proton therapy lowers the risk of secondary cancers and other radiation‐induced complications later in life.

However, the benefits of proton therapy rely heavily on accurate beam delivery. The precision required for proton therapy means that even slight deviations in spot position, size, or isocentricity can result in significant discrepancies in the dose delivered to the tumor or surrounding tissues.^5^ Such inaccuracies can compromise treatment effectiveness, potentially leading to inadequate tumor control or increased harm to healthy tissue. Therefore, rigorous quality assurance (QA) is essential to ensure the proton beam is delivered exactly as planned.^6^, ^7^ Accurate beam delivery is not just a technical requirement—it is critical for maximizing the therapeutic benefits of proton therapy while minimizing patient risk.

Over the years, various studies have emphasized the importance of QA in proton therapy, employing different methodologies. For instance, Christian et al. introduced a comprehensive quality assurance program for the German Heavy Ion Therapy Project, incorporating ion‐specific tests and standard protocols to ensure consistent treatment quality and safety during commissioning and clinical operation.^8^ Arjomandy et al. described QA processes at the MD Anderson Proton Therapy Center, with an emphasis on routine testing, but without specific details on spot scanning systems.^9^ Further advancements in QA methodologies have been reported by Mirandola et al., who detailed QA processes for actively scanned proton and carbon ion beams, using Gafchromic films for daily spot position and spot size checks.^10^ Hartmann et al. and Farr et al. explored alternative QA tools like flat‐panel detectors^11^ and the Lynx detector (IBA Dosimetry, Schwarzenbruck, Germany),^12^ demonstrating their effectiveness for spot verification. Chen et al. (2016) further advanced QA practices by using a phosphor screen with a CCD camera for monthly QA, emphasizing the importance of precise alignment and advanced software for data analysis.^13^ Tan et al. presented a method for measuring dispersion in proton therapy accelerators by assessing spot position and momentum deviations within a spill, emphasizing its role in minimizing dose fluctuations and recommending its inclusion in QA protocols.^14^ In another study, a scintillator and 2D array detector were used to measure spot size and position for the first compact proton therapy system with spot scanning and dynamic field collimation.^15^ Almurayshid et al. developed a proton therapy QA system using a plastic scintillator and a camera, addressing dose reproducibility, dose‐response linearity, and quenching at the Bragg peak with Birks' law. Their system demonstrated accuracy and was validated through Monte Carlo simulations, making it suitable for clinical QA.^16^

Building on previous work, this study introduces a novel QA framework for the Hitachi Probeat‐V Proton Beam Therapy System, which features a 190° half‐gantry and a couch with 180° rotational capability. In this system, one monitor unit (MU) corresponds to approximately 10 000 counts from a 1%‐precision analog‐to‐digital converter, reflecting collected charge in the main dose monitor; its relation to dose depends on spot energy and location.^17^ The spot size in air at the range shifter varies from 5.4 to 2.3 mm across energies from 71.3 to 228.8 MeV. To assess gantry and couch isocentricity, spot position accuracy, and spot size stability, the framework combines a custom‐designed Octopoint phantom with Gafchromic EBT3 film analysis. Using the high spatial resolution of Gafchromic film^18^ along with spot position monitor (SPM) log file analysis, the framework provides a comprehensive assessment of treatment accuracy. Designed to enhance the precision and reliability of proton therapy delivery, this approach is adaptable to various clinical quality assurance tasks, advancing QA processes, and improving patient outcomes and safety.

## 2 MATERIALS AND METHODS

### 2.1 End‐to‐end gantry and couch isocentricity

We have developed a comprehensive end‐to‐end test that integrates our clinical record‐and‐verify system (R&V) with image guidance for accurate phantom positioning. Inspired by the Winston–Lutz method initially designed for Linac‐based radiosurgery,^19^ this approach focuses on irradiating a ball‐bearing (BB) positioned at the isocenter. Fields are delivered using various combinations of gantry and patient positioning system (PPS) yaw angles. This test is particularly important given the need to correct for virtual isocenter (x, y, z) alignment, especially in our half‐gantry system.^20^ In this system, the heavy weight of the gantry causes the isocenter position to shift as the gantry angle changes. Virtual isocenter correction compensates for these shifts by adjusting the PPS, ensuring accurate alignment and treatment delivery.

#### 2.1.1 Design of the Octopoint phantom

**FIGURE 1:**
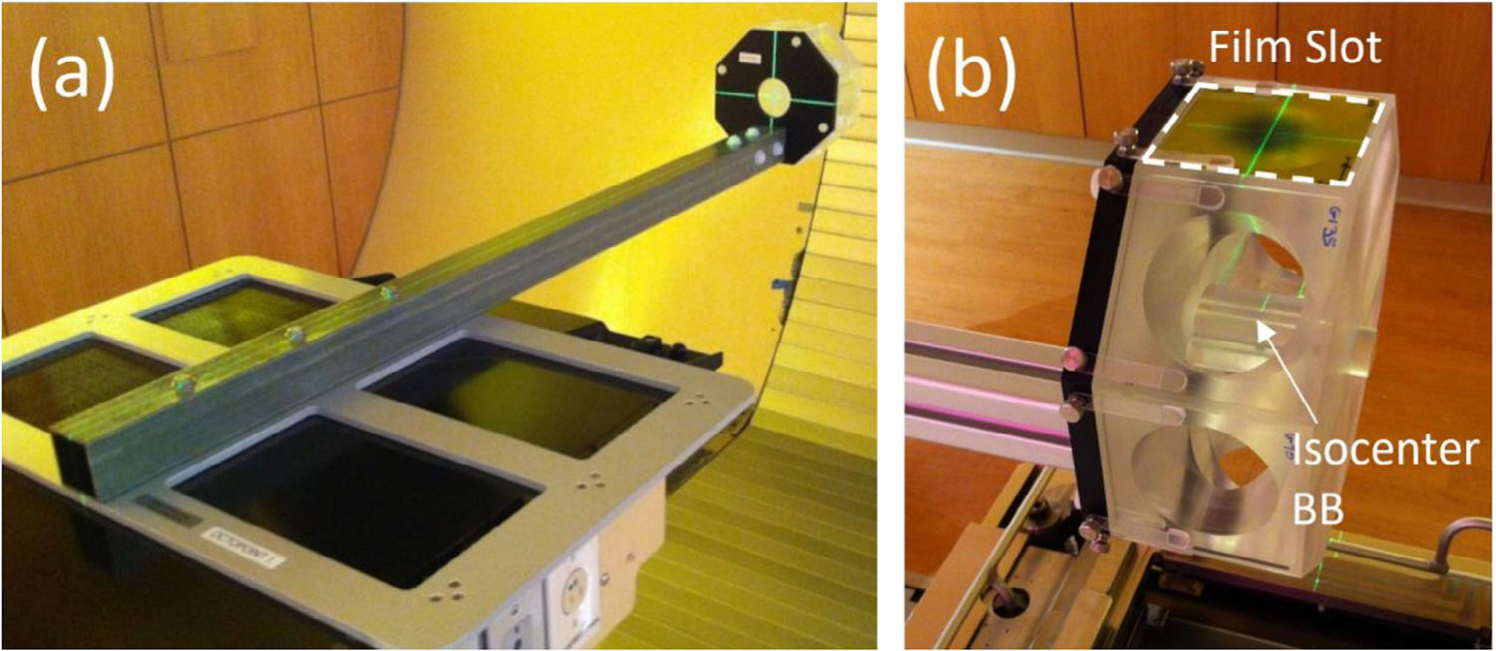
(a) The Octopoint device positioned in one of our spot‐scanning proton therapy gantry rooms, indexed to the treatment couch (Civco Universal Couch Top with extension removed). (b) Close‐up of the Octopoint phantom, highlighting one of the eight film slots within the dashed rectangle, with a white arrow indicating the isocenter BB. BB, ball bearing.

The Octopoint phantom is machined from a solid octagonal acrylic block, originally measuring 176^2^ mm^2^ × 69 mm, and is secured to the PPS couch base using a fiberglass post and index frames (Figure 1). To avoid interference with imaging quality, no metal is used in attaching the phantom to the support post. Its geometric design allows irradiation from gantry angles in 45‐degree increments, accommodating any PPS yaw angle for anterior or posterior beams.

At its core, the phantom houses interchangeable cylindrical cartridges, each containing a high‐precision steel ball bearing (Isocenter BB) with diameters of 2, 3, and 5 mm. A blank acrylic cartridge is available for artifact‐free CT scanning, though scanning with the 2 mm isocentric BB still produces an acceptable level of scatter artifact, enabling accurate isocenter placement in treatment planning. Since proton beam spot sizes vary with energy, the use of different‐sized isocenter BBs is essential for evaluating system isocentricity across the energy range of 71.3–228.8 MeV.

Each face of the octagon holds Gafchromic film (≤65 × 65 mm^2^) positioned 88 mm distal to the BB. These film slots correspond to various couch and gantry angle combinations, with arrows indicating the insertion direction. Four small metallic fiducials embedded around the device's periphery facilitate image guidance and six‐degree‐of‐freedom (6DOF) positional corrections while remaining clear of the beam paths. Additionally, painted scribe marks on the transverse, sagittal, and coronal planes aid in verifying room‐laser alignment after image guidance, ensuring consistent positioning throughout the process.

#### 2.1.2 Isocentricity test workflow using the Octopoint phantom

For routine monthly QA testing using the Octopoint protocol, the workflow begins by securing the phantom to the couch, which is initially positioned at an arbitrary PPS (x, y, z) location within the field of view of the positioning image analysis system (PIAS). The PIAS includes floor‐mounted x‐ray tubes and ceiling‐mounted imaging panels that capture paired orthogonal x‐ray images. The PPS yaw angle is set to 270° (the nominal couch angle for imaging), and the gantry is positioned at 90°. Additional weight is added to the couch to simulate realistic patient conditions. The gantry angle of 90° is crucial, as it serves as the reference for laser calibration and is integral to the virtual couch corrections used for proton delivery accuracy, which depend on the gantry angle.

Next, the treatment plan is sent from the R&V system. PIAS imaging is performed, followed by automatic 6 DOF matching and application of the derived shifts. Fiducial positions, particularly the isocentric BB, are verified, and any necessary PPS adjustments are made. Residual (x, y, z) translation errors of the isocentric BB are corrected manually, with confirmation through further PIAS imaging.

**FIGURE 2:**
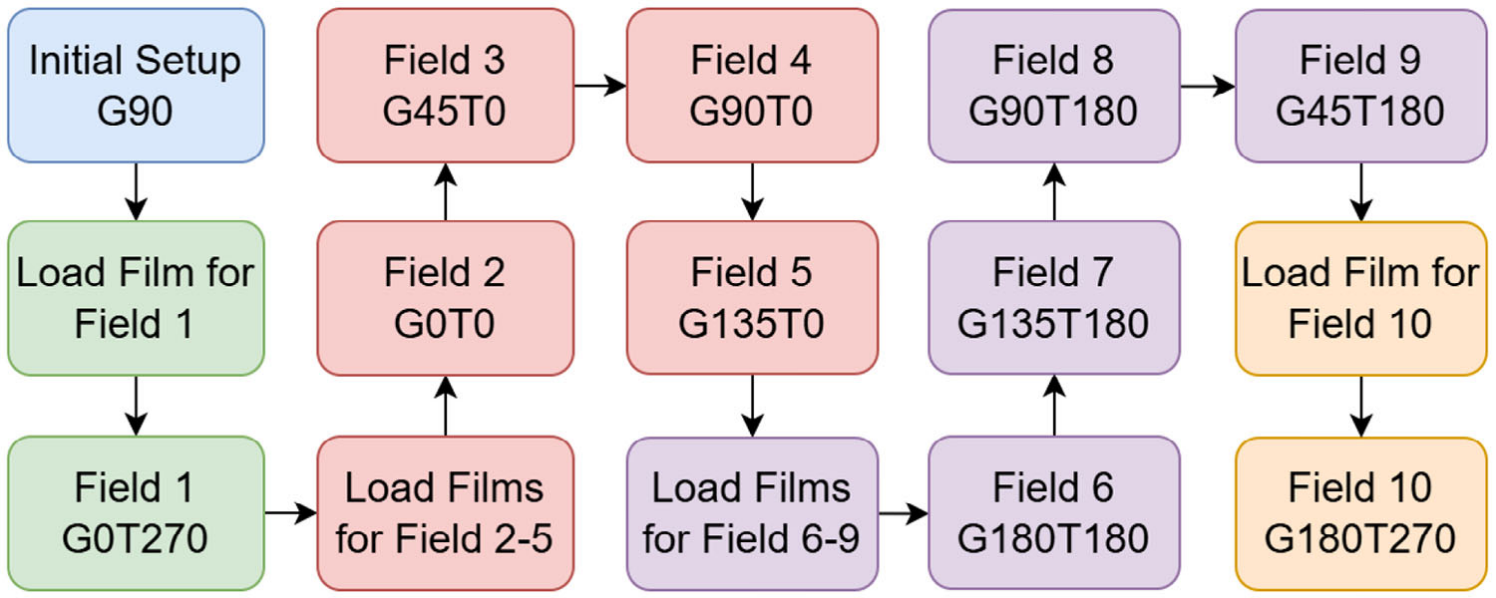
Flowchart of the steps involved in the monthly isocentricity test. Each field is identified by its gantry angle (G) and couch angle (T).

Once the Octopoint phantom is precisely positioned, Gafchromic films are sequentially loaded into the eight film slots on the phantom. These films are irradiated using 10 different combinations of gantry and couch angles, as detailed in Figure 2. Films that do not overlap in dose can be loaded and removed simultaneously. To reflect clinical practice, instead of a single high‐MU spot at the center (x = 0 cm, y = 0 cm), each field is irradiated with multiple clinically relevant MU/spot. Specifically, each film receives 5 MU distributed across 1000 spots, equating to 0.005 MU per spot. To complement the film analysis, SPM log data (see Section 2.2.3) is recorded for fields 2–6, providing a reference measure of spot position accuracy for center spots (x = 0 cm, y = 0 cm) over the gantry angles tested (0°, 45°, 90°, 135°, and 180°). The selected beam energy of 228.8 MeV matches the center spot energy used in the spot position accuracy test described later.

Additionally, the monthly Octopoint protocol includes a test of the combined PPS calibration and PIAS imaging geometry consistency. If the PIAS imaging match is accurate and the PPS is functioning properly, the final couch position, after aligning the Octopoint phantom with the plan, should remain consistent each month. To confirm this, each gantry room uses a designated PPS couch baseline to check the final (x, y, z) coordinates following image guidance.

#### 2.1.3 Octopoint Gafchromic film analysis

Film analysis follows our established institutional protocols.^21^ Exposed Gafchromic films receive a peak dose of 5–10 Gy, typically deposited in the plateau region of the proton depth–dose curve. The films are scanned at 200 dots per inch (dpi) using a flatbed scanner (Epson 1100XL, Epson Inc.). To maximize efficiency, five films are scanned simultaneously, positioned adjacent to one another, and centered laterally within the scanning area. Alignment is maintained using a thin cardboard guide, and tempered glass is used to eliminate any air gaps between the films and the scanner surface. Dose conversion is performed using FilmQA Pro (Ashland, Inc.), with lot‐specific calibrations based on MV X‐ray irradiation. All films are scanned in the same orientation, with the scanner's long axis aligned with the short dimension of the pre‐cut films. Analysis is performed using the average dose across all color channels.

**FIGURE 3:**
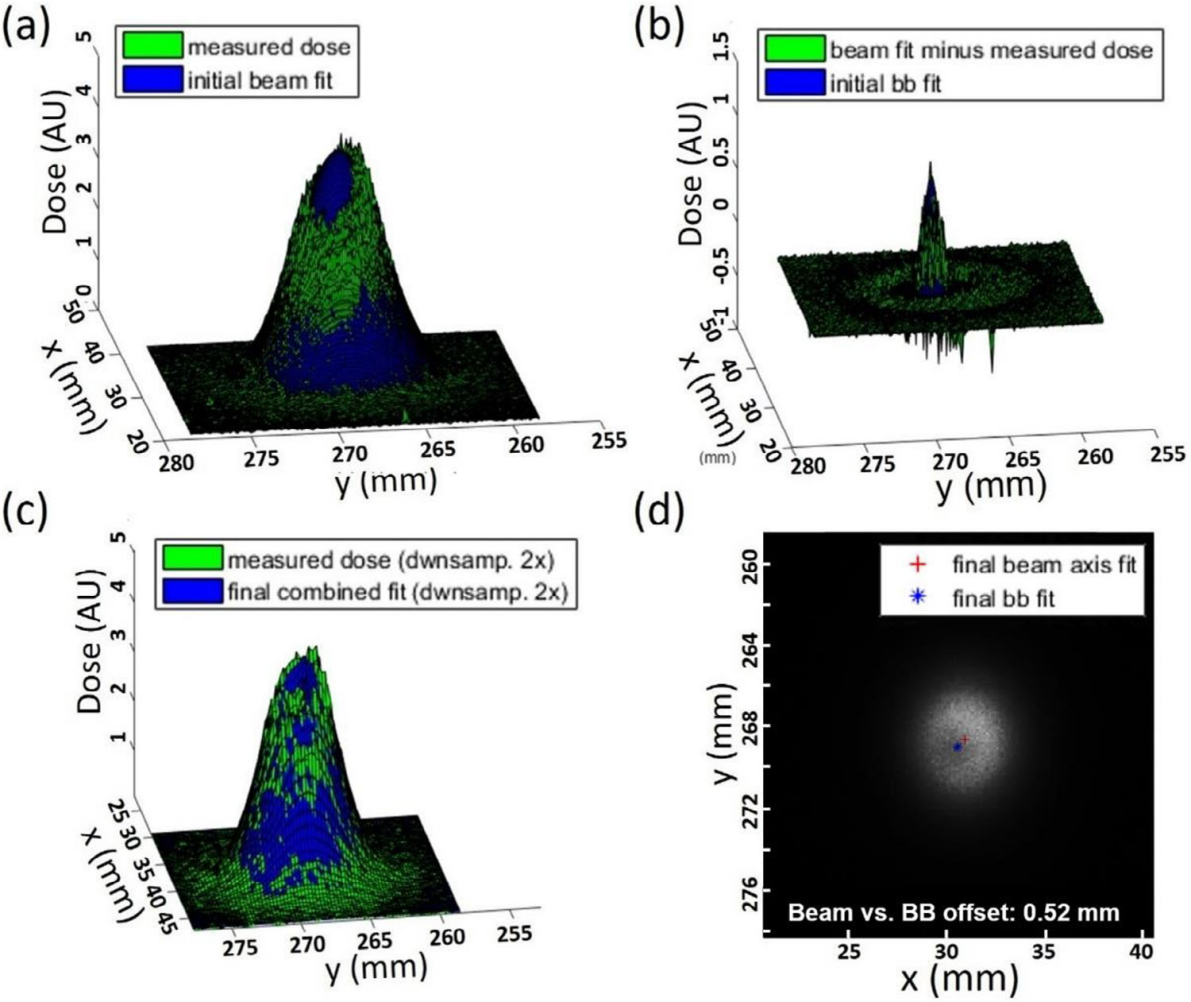
Stepwise fitting process for dose‐converted films. (a) Initial 2D Gaussian fit ignoring BB perturbation. (b) Subtraction of measurement from the initial fit to approximate the centroid and sigma for the BB perturbation. (c) Final 11‐parameter fit, seeded with initial parameters. d) Result showing final beam and BB centroid locations. BB, ball bearing.

An in‐house processing routine and graphical user interface (GUI) were developed using MATLAB (The MathWorks Inc., Natick, Massachusetts, USA) to analyze the 2D dose distributions from the irradiated films. This tool processes TIFF files for each gantry/couch position and the corresponding machine log files to correlate the film plane with the isoplane. The analysis assumes an asymmetric 2D Gaussian for the beam convolved with a symmetric 2D Gaussian for the BB perturbation, requiring 11 fitting parameters to accurately determine BB‐beam offsets for each film. Figure 3 illustrates the step‐by‐step 2D fitting process used to analyze observed radiation profiles, designed for robustness. In Figure 3a, the blue 3D volume represents the initial asymmetric 2D Gaussian fit of the beam, excluding any BB perturbation, while the green 3D volume shows the dose measured using film. In Figure 3b, the green volume represents the difference between the fitted beam profile and the measured dose, which was then used to generate an initial symmetric 2D Gaussian fit for the BB, shown in blue. Figure 3c compares the film‐measured dose with the combined profile of the beam and BB fits; in this final optimization, all fitting parameters are allowed to vary. Finally, Figure 3d shows the output from the MATLAB code, displaying the final beam axis fit and BB fit, with the absolute offset between them calculated. In this example, as in the routine monthly tests, the selected beam energy was 228.8 MeV, and the isocentric BB used was 2 mm in diameter. The commissioning process for the Octopoint data analysis involved testing beams at low, medium, and high energies. Results showed that Octopoint performed consistently well across all clinically used energies at our facility. Based on these findings, we recommend using 5, 3, and 2 mm isocentric BBs for low, medium, and high‐energy beams, respectively.

#### 2.1.4 Repeatability and sensitivity analysis

To evaluate system reproducibility, the end‐to‐end isocentricity test was conducted three consecutive times in a single vault. Each iteration began with a complete reset, including the physical removal and re‐setup of the Octopoint phantom, followed by repeated image guidance for accurate positioning.

A detailed sensitivity and accuracy test was also performed to quantify the system's ability to detect deviations in isocentricity. After standard image‐guided radiation therapy (IGRT) setup using the Octopoint phantom, known shifts were introduced to the PPS. These shifts were applied independently along the positive (x, y, z) axes in increments of 0.5 mm (+0.5, +1.0, +1.5 mm). The full Octopoint film irradiation procedure was then performed to assess the system's response. This test was designed to establish appropriate warning and action thresholds for our QA program.

### 2.2 Spot position accuracy and spot size constancy

In addition to the absolute targeting accuracy evaluated by the Octopoint test, it is crucial to maintain spot position accuracy across the treatment field and ensure the stability of beamline optical tuning, which directly affects spot size consistency. To address these needs, we developed a multi‐energy spot position accuracy and spot size constancy test using Gafchromic films, focusing on ease of setup and sensitivity to deviations from the baseline. We later enhanced this test by incorporating logged SPM data to establish baselines and compare spot‐by‐spot measurements with our film results. This addition was driven by our increasing reliance on SPM log data for patient‐specific quality assurance.^22^

#### 2.2.1 Spot size and position film measurements

**FIGURE 4:**
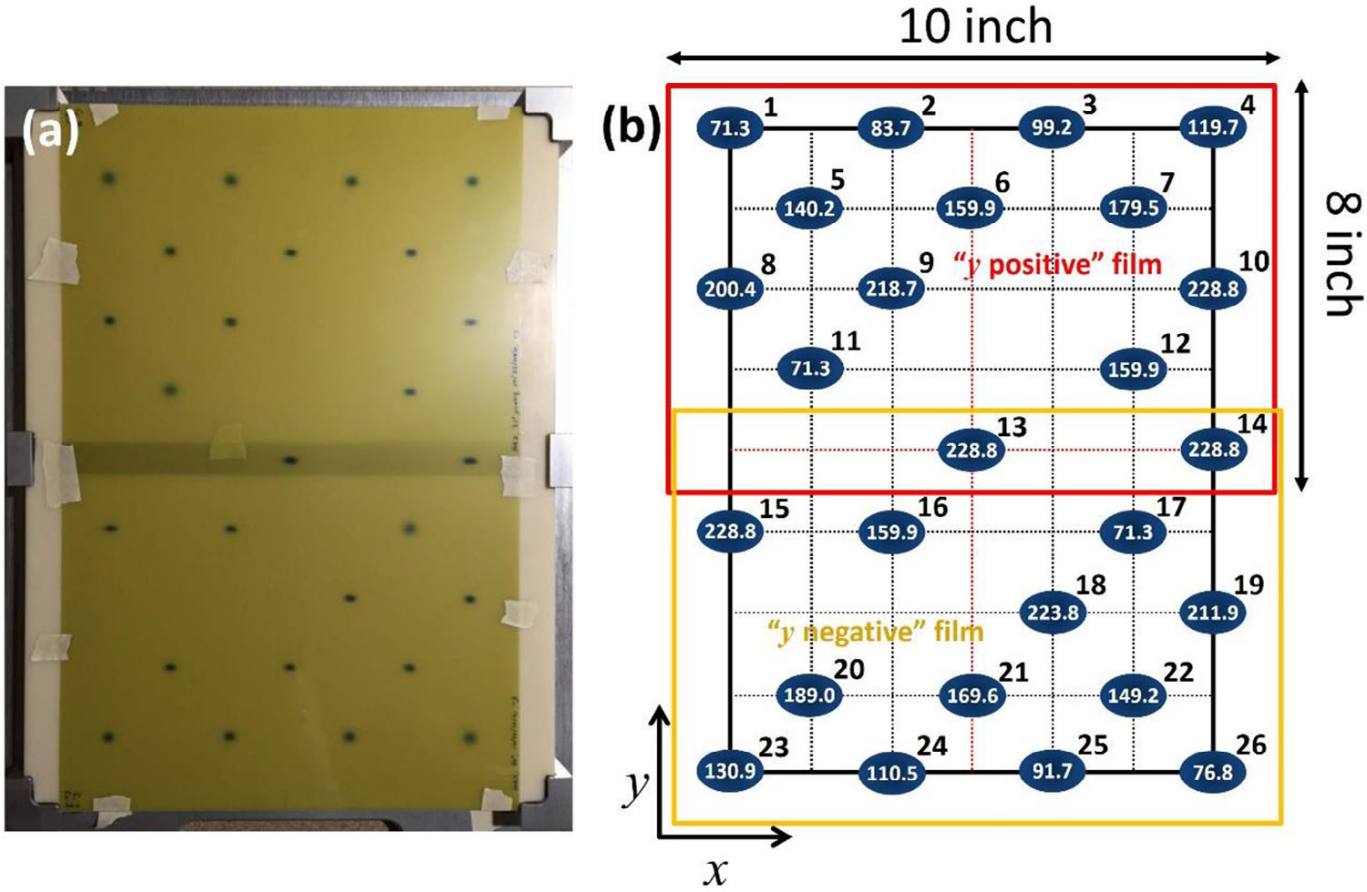
(a) Two overlapping 8″ × 10″ Gafchromic films are taped to the upstream side of the range shifter, aligned by the outer edges of both the film and the range shifter. (b) Illustration of spot placement in nozzle coordinates and corresponding energy per spot (MeV) for the spot position and size test (spot sizes are not to scale). The grid spacing, projected at the isocenter plane, is 5 cm, covering the full deliverable field size of 30 cm × 40 cm.

Figure 4a shows the setup where two 8″ × 10″ sheets of Gafchromic films are attached to the upstream side of a 2.5 cm range shifter, positioned approximately 45 cm upstream of the isocenter. The films are taped along their edges to secure them in place, with an overlap near y = 0 cm (the nozzle scanning coordinate). Together, the films cover the entire field size, spanning 30 cm in the *x*‐dimension and 40 cm in the *y*‐dimension of the nozzle, without needing to be cut.

The spot pattern, consisting of 26 locations, is arranged on a non‐symmetric grid across the 30 cm × 40 cm^2^ field, as shown in Figure 4b. The grid has a nominal 5 cm spacing. The central group, within |y| ≤ 5 cm of the nozzle, includes “S3” energies, representing the lowest (71.3 MeV), medium (159.9 MeV), and highest energy levels (228.8 MeV). “S9” energies, which benchmark across the available energy range, are positioned at *y* ≥ 10 cm, while nine additional energies are placed at *y* ≤ −10 cm: 71.3, 76.8, 83.7, 91.7, 99.2, 110.5, 119.7, 130.9, 140.2, 149.2, 159.9, 169.6, 179.5, 189.0, 200.4, 211.9, 218.7, 223.8, and 228.8 MeV. The overlap region between the films effectively captures two 228.8 MeV spots at *y* = 0 cm on both films.

Each spot location contains 100 discrete spots, delivering 0.01 MU per spot, for a total of 1 MU per spot location. Peak doses measured in the films typically range between 5 and 12 Gy per spot location. This test is currently performed at gantry angles of 0°, 90°, and 180°.

We selected a large dose to create a clear optical density impression on the film, making both spot position and Octopoint measurements easily visible to the eye. The total MU was subdivided into 0.01 MU per spot to better align with the typical patient MU per spot. This approach also takes into account control system factors, such as systematic spatial drift during spot extraction (which is caused by the “RF Kicker's” increasing influence on proton beams phase space distribution). Additionally, this alignment with typical clinical MU/spot helps to incorporate relevant fluctuations in spot position, size, and intensity caused by factors such as mechanical tolerances, magnet stability, and beam current variations. By delivering and averaging over 100 spots, these random variations are minimized, allowing for a more accurate and reliable assessment of the system's true performance at the measurement location.

#### 2.2.2 Spot size and position film analysis

For spot size and position analysis, we developed an in‐house MATLAB tool to determine the raw film coordinates of all measured spots. Two Gafchromic films, labeled “*y*‐positive” and “*y*‐negative,” are scanned simultaneously on a flatbed scanner at 150 dpi. The films are aligned so that the nozzle *x* coordinates match the scanner's lateral orientation, consistent with dose calibration procedures. A clean glass plate is placed over the films during scanning, and dose conversion is performed using FilmQApro.

Spot locations on the films are identified by fitting them with a 2D asymmetric Gaussian function. Initial centroids are estimated using a templated set of locations, which can be confirmed or adjusted via a GUI. The spot positions are then refined within a 15 × 15 mm^2^ region of interest using a fitting algorithm. Spot sizes at the 2.5 cm range‐shifter position are tracked by recording the major and minor axis widths of the 2D Gaussian fit, compared to a baseline dataset established during gantry commissioning.

Raw spot coordinates are exported to Microsoft Excel for semi‐automated analysis. The center spot of 228.8 MeV on the *y*‐positive film, validated in the end‐to‐end Isocentricity test described in Section 2.1, is assumed to be at the origin (*x* = 0, *y* = 0). To optimize alignment of spots within each row (at isocenter coordinates of *y* = +5, +10, +15, and +20 cm), Excel's “Solver” function is used to adjust the coordinate axes of the *y*‐positive film around its center spot. This ensures consistent *x* and *y*‐coordinate values for spots with the same planned positions, correcting for minor alignment errors caused by film positioning on the range shifter or scanner. This optimization typically involves a small rotation angle, closely monitored to trigger a warning if the most distant spot shifts by more than 1 mm (equivalent to 0.229°). An alarm is set for shifts exceeding 1.5 mm (0.344°), based on Hitachi's spot position accuracy specifications. The *y*‐negative film is similarly translated and rotated to match the *y*‐positive film's coordinates, ensuring alignment within the same thresholds for consistency.

**FIGURE 5:**
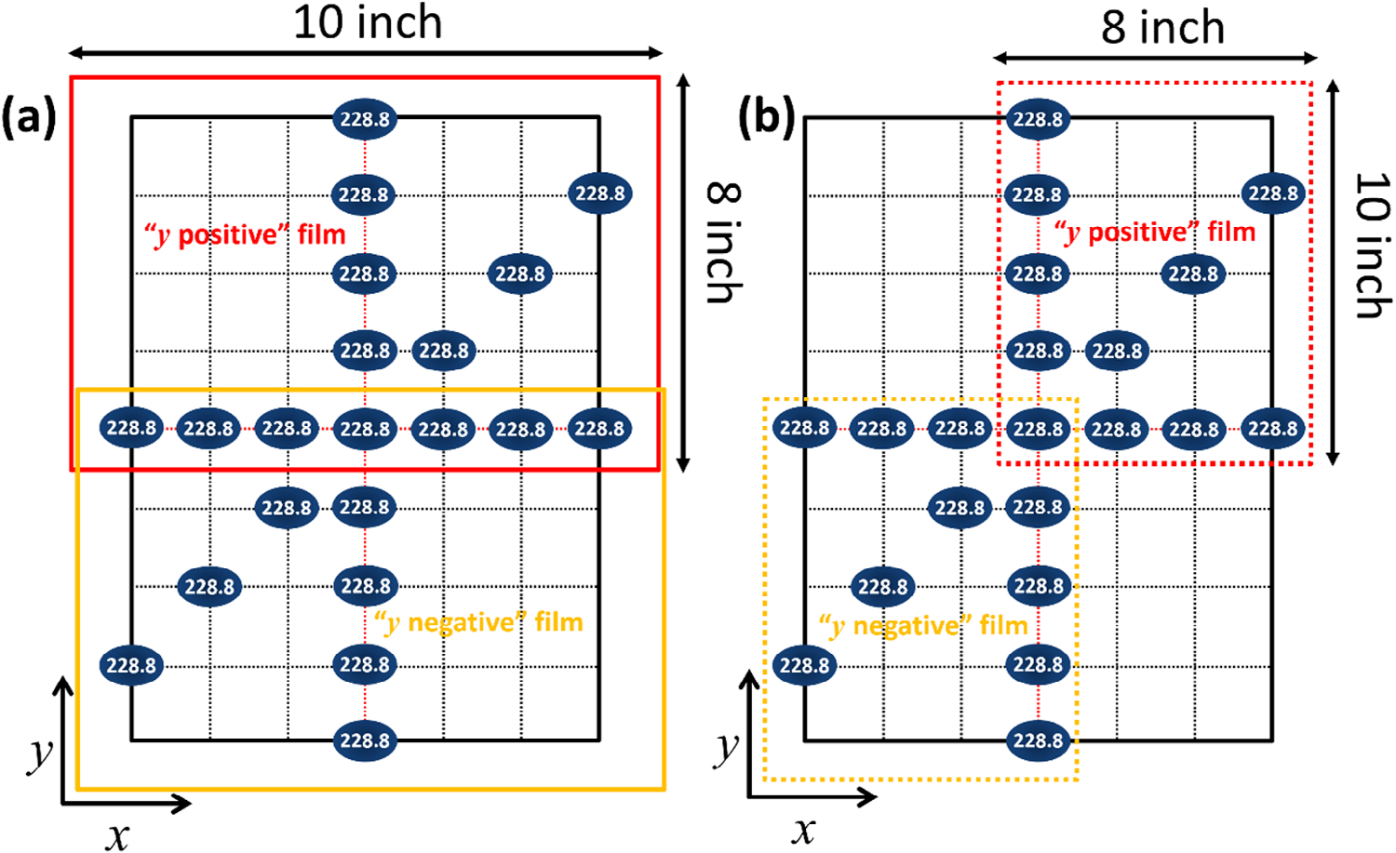
Spot placement in nozzle coordinates and energy per spot (MeV) for the spot position and size “divergence commissioning” process, shown at (a) the 2.5 cm range shifter and (b) the isocenter plane (spot sizes are not to scale).

Using the range shifter as a holder for Gafchromic film offers several benefits, including a straightforward setup across various gantry angles and minimal additional equipment requirements. However, since measurements are taken upstream of the isocenter plane, interpreting spot position and size data becomes more complex. To overcome this, we developed a divergence correction strategy to project spot positions measured at the 2.5 cm range shifter onto the isocenter plane. This correction process begins with a commissioning step that involves simultaneous irradiation of two sets of films at a monoenergetic energy of 228.8 MeV—one positioned at the 2.5 cm range shifter (Figure 5a) and the other at the isocenter plane (Figure 5b). The films at the range shifter are arranged similarly to those in the spot size and position test, with two overlapping 8″ × 10″ films taped to the range shifter. At the isocenter plane, irradiation patterns are applied to both the positive quadrant (*x*, *y*) and the negative quadrant (−*x*, −*y*) of the nozzle coordinates.

In developing this strategy, we assumed that the effective points of beam divergence in the nozzle's *x* and *y* directions can vary between gantry rooms and angles but remain independent of beam energy. During analysis, which aimed to minimize the average (*x*, *y*) errors between actual and projected spots, we treated the effective points of beam divergence in both the *x* and *y* directions as variables specific to each gantry room and angle. The distance from the upstream film to the isocenter plane was fixed based on known geometric factors.

#### 2.2.3 SPM log file analysis

The vendor provides a capability to log data recorded by the SPM in .csv format on a per‐spot basis from the gantry console computer. The SPM logs include measurements of spot position in nozzle coordinates (*x*, *y*) and projections of spot widths along the same axes (σ_SPM,x_, σ_SPM,y_). This data enables us to: (1) monitor SPM‐derived spot position and width relative to baseline measurements, (2) compare SPM‐derived spot positions (adjusted to the isocenter plane using vendor‐supplied divergence correction coefficients) with independent film‐based spot position measurements, and (3) potentially assess beam tuning stability by comparing spot widths at the SPM plane with those measured on film.

Expanding on the third point, without the SPM detector itself, spot width measured at the SPM plane tends to decrease as it approaches the isocenter due to beam optics or tuning (e.g., quadrupole magnets used for focusing). However, factors such as multiple Coulomb scattering (MCS) within the SPM (including gas, sensitive elements, and exit windows), along with air scatter, contribute to an enlargement of the spot width as the beam travels toward the isocenter. These effects counteract, or are convolved with, beam optics. Based on Moliere's MCS theory for a thin target, the angular spread (θ₀) is proportional to 1/β^2^, where β represents the beam velocity as a fraction of the speed of light.^2^

Assuming the beam‐focusing effect depends linearly on distance from a downstream focal point, we postulate that the spot width at the 2.5 cm range shifter position (σ_RS_) can be expressed as:

$$\sigma_{\mathrm{RS},(x,y)}^{2}={\left[\left(F\left(\beta \right)-L\right)*\sigma_{\mathrm{SPM},(x,y)}/F\left(\beta \right)\right]}^{2}+{\left[B*L/\beta^{2}\right]}^{2}$$

The first term relates to tuning, describing how the beam focuses toward the isocenter, with F(β) representing an energy‐dependent focal length parameter, L as the constant distance from the SPM to the 2.5 cm range shifter (approximately 81 mm), and β the beam velocity. The second term accounts for MCS effects, approximating the spot size increase as a function of beam energy (velocity), with B as an empirically derived fitting coefficient.

Herein, we aim to provide a proof‐of‐concept, potentially offering a means to monitor (vendor‐controlled) aspects of beam performance (beam optics) per vault. Partly for convenience, we assume no energy dependence of F(β), F(β)→F, treating it as a constant. This is not unreasonable under the assumption that the vendor has tuned the beam optics, per energy, to minimize spot position at the isocenter plane.

#### 2.2.4 Spot position test sensitivity analysis

We conducted sensitivity testing to evaluate clinically relevant deviations and their impact against established warning and alarm thresholds. In the first set of tests, we introduced 1.0 mm shifts to spot positions under two scenarios. In the first scenario, the center spot was shifted by +1 mm in both the *x* and *y* directions. In the second scenario, shifts were applied to random spots in each quadrant of the (*x*, *y*) plane. Specifically, Spot 1 in Quadrant 1 (71.3 MeV) was shifted by +1 mm in the *x* direction, Spot 7 in Quadrant 2 (179.5 MeV) was shifted by −1 mm in the *x* direction, Spot 23 in Quadrant 3 (130.9 MeV) was shifted by +1 mm in the y direction, and Spot 19 in Quadrant 4 (211.9 MeV) was shifted by −1 mm in the *y* direction. In the second set of tests, we simulated a 1% gain or calibration error in the SPM. This error was modeled by uniformly reducing the *x* and *y* positions of all spots by 1% of their nominal values.

## 3 RESULTS

### 3.1 End‐to‐end gantry and couch isocentricity

At our institution, we have four gantry‐based treatment vaults, commissioned and brought into clinical operation over approximately one year. Gantries 3 and 4 (G3 and G4), located furthest from the accelerator, were commissioned first, followed by Gantry 2 and 1 (G2 and G1). To assess the isocentricity of each gantry and establish baseline data, we monitored Octopoint isocentricity test results from the initial clinical release of each vault through December of the following year, collecting at least 8 months of data for each.

**FIGURE 6:**
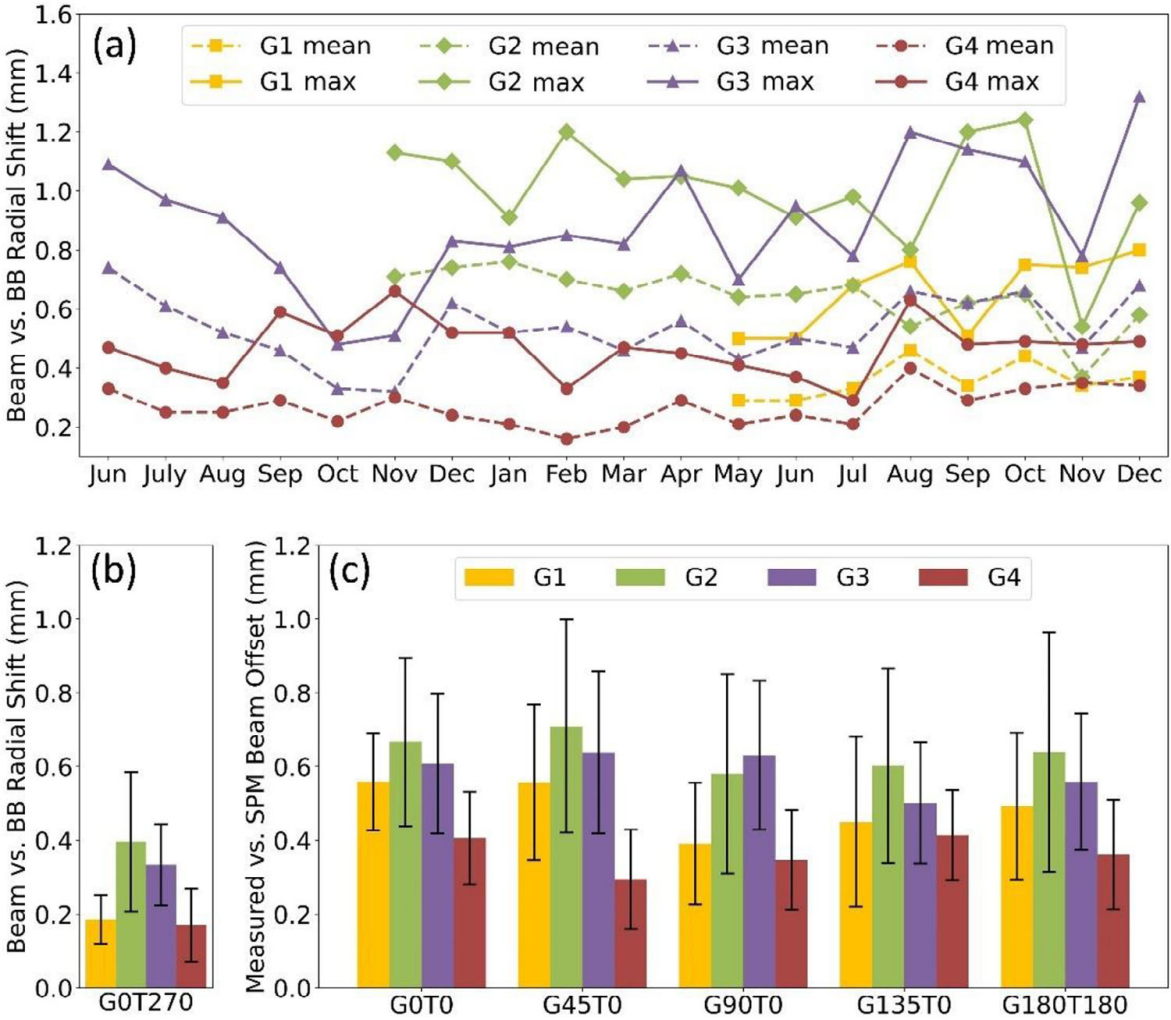
End‐to‐end gantry isocentricity testing using Octopoint for four gantries. (a) Octopoint‐measured beam versus BB radial offsets for each gantry room, with dashed lines representing mean values and solid lines indicating maximum values. (b) Octopoint‐measured beam versus BB radial offsets for each gantry room, focusing on the first beam in the protocol, delivered immediately after IGRT (G0T270). (c) Comparison of Octopoint‐measured versus SPM‐recorded beam offsets for a subset of gantry and table‐angle combinations. The bars in plots (b) and (c) represent the standard deviation of the data. BB, ball bearing; SPM, spot position monitor.

Figure 6a shows the monthly trends in isocentricity, displaying the mean and maximum beam‐to‐isocentric BB offsets (measured as radial distance in mm) for the beams used in the test protocol. Figure 6b summarizes the radial beam‐to‐BB offset results for each gantry, focusing on the first beam in the protocol, which is delivered immediately after IGRT. Figure 6c highlights the discrepancy in radial distance between the beam position measured by Octopoint and the position recorded by the SPM, which provides a vendor‐supplied record of spot positions. Currently, SPM data is recorded for test fields 2–6 (G0T0, G45T0, G90T0, G135T0, G180T180).

In the Octopoint repeatability test, we evaluated the radial offsets between the Octopoint‐measured beam and the BB across three consecutive measurements. This assessment was conducted for 10 different couch‐gantry combinations and demonstrated a maximum standard deviation of 0.1 mm.

In the Octopoint sensitivity test, the warning threshold was set at 1 mm, and the failure threshold at 1.5 mm. These settings were informed by our vendor's overall isocentricity specifications. Sensitivity was quantified by analyzing the number of warnings and failures observed across 10 films under various error scenarios. These scenarios involved intentional shifts of 0.5 mm (within tolerance), 1 mm (at warning level), and 1.5 mm (at failure level) along three different axes. The results are summarized in Table 1, which presents a statistical overview of the warnings and failures for each shift condition. For instance, with an applied shift of 1.5 mm along the *Z*‐axis, 4 out of 10 measured films showed a warning, while none registered a failure. It is worth noting that warnings are rarely observed during routine measurements, and even a single warning can indicate potential issues with runout.

**TABLE 1:** Number of warnings and failures among 10 repeated measurements for each applied Octopoint shift along the *X*, *Y*, and *Z* axes. “Warnings Only” refers to deviations between 1.0 and 1.5 mm. “Fails Only” refers to deviations greater than 1.5 mm.

Axis	*X*			*Y*			*Z*		
Applied Octopoint Shift (mm)	0.5	1.0	1.5	0.5	1.0	1.5	0.5	1.0	1.5
Number of Warnings Only (1.0 mm < error ≤ 1.5 mm, 10 measurements)	2	4	3	1	9	1	0	2	4
Number of Fails Only (error > 1.5 mm, 10 measurements)	0	1	4	0	0	9	0	0	0

### 3.2 Spot position accuracy and spot size constancy

**FIGURE 7:**
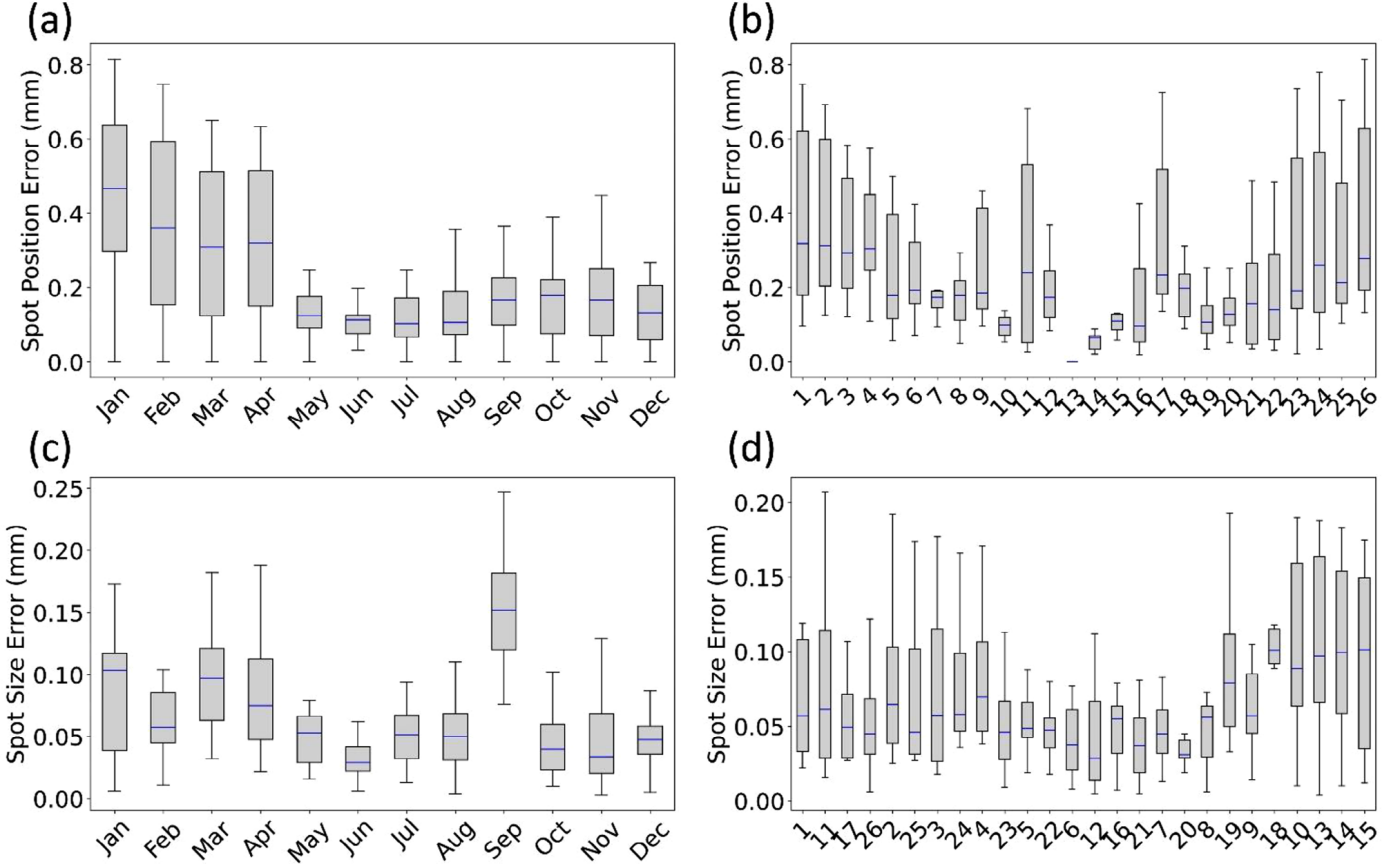
Year‐long monitoring of spot position and spot size deviations for a specific gantry. (a) Spot position error across all spots over the initial year surveyed. (b) Spot position error for each individual spot, excluding the center spot, over the initial year surveyed. (c) Spot size error across all spots over the initial year surveyed. (d) Spot size error for each individual spot, including the center spot, over the initial year surveyed, X‐label is sorted based on energy. The spot numbers in (b) and (d) correspond to the pattern shown in Figure 4.

Figure 7 presents the film‐based measurements of deviations in spot position at the isocenter plane and spot size at the 2.5 cm range shifter plane, tracked over one year for Gantry 3. These measurements were obtained using a 26‐spot, multi‐energy pattern delivered across a 30 cm × 40 cm field (as shown in Figure 4). As part of our monitoring program, spot position and size deviations were calculated relative to baseline film measurements. Spot size error is expressed using the metric Δσ, calculated as (Δσ_major_^2^ +Δσ_minor_^2^)^1/2^, representing the combined deviations along the major and minor axes of the 2D ellipse. In the figure, the top row illustrates spot position error, while the bottom row displays spot size error. Each row includes two components: the overall error across all spots over time and the individual error for each spot over the 1‐year period. The boxes represent the interquartile range (25th–75th percentile) of the spot position/size errors, with blue lines indicating the medians; whiskers extend to the nearest data points within 1.5 times the interquartile range, beyond which data are considered outliers. In Figure 7b,d, the *x*‐axis corresponds to the spot numbers identified in Figure 4. Notably, the center spot (Spot 13: 228.8 MeV) is excluded from the spot position error plots (Figure 7b) since it is set at the origin in the analysis but is included in the spot size error analysis (Figure 7d).

**FIGURE 8:**
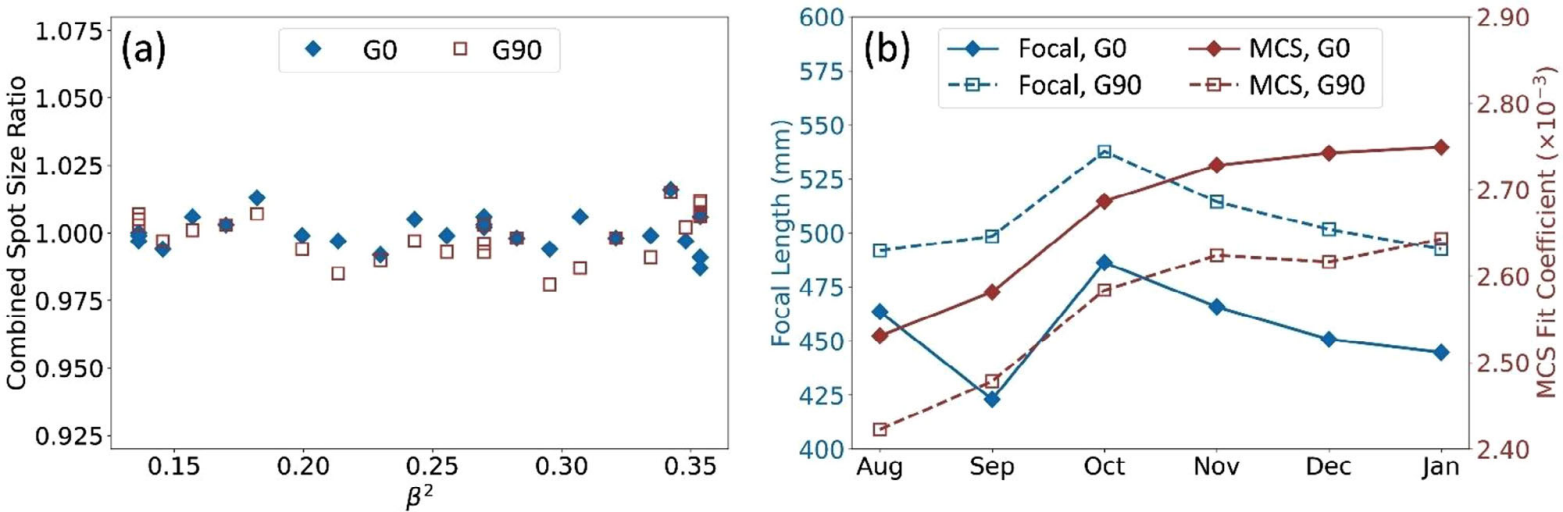
(a) Combined spot size ratio plotted versus the β^2^ for a single vault at gantry angles of 0° and 90° for the 26 spots included in the test. There are multiple dots at the same β^2^ because of repeated energy in the pattern. (b) Trends for the two fitting parameters (focal length and MCS coefficient, respectively) per gantry room and gantry angle. MCS, multiple Coulomb scattering.

Figure 8a compares SPM‐log and film‐measured spot sizes for a specific gantry at couch angles of 0° and 90°, obtained during a monthly spot size test. The plot shows combined spot size ratios as a function of β^2^ (β  = v/c), where β = 0.1363 corresponds to the lowest energy (71.3 MeV) and β  = 0.3537 represents the highest energy (228.8 MeV) in the 26‐spot pattern. These combined spot size ratios are calculated by dividing the convolved spot size measured by film (σ_major_^2^ +σ_minor_^2^)^1/2^ by the predicted spot size at the film location (at the 2.5 cm range shifter) derived from SPM data (σ_RS,x_^2^ +σ_RS,y_^2^)^1/2^. An ideal fit would result in a combined spot size ratio of 1, indicating that the predicted spot size at the range shifter based on SPM data matches the measured spot size. The predictions depend on two key parameters: focal length (F) and the MCS coefficient (B). These parameters are optimized monthly by minimizing the sum of squared differences between the convolved spot sizes for the 26 spots, specific to each gantry and gantry angle. Figure 8b tracks the change in these optimized fit parameters over a six‐month period for G3.

This test is sensitive to small changes from a defined baseline. When shifting the center spot by 1 mm along the *x*‐axis, film measurements for the other spots consistently showed a shift of 1.02 mm (± 0.29 mm). Similarly, for a 1 mm shift along the *y*‐axis, the measured shift was 1.02 mm (± 0.23 mm). For randomly selected spots shifted by 1 mm, Spot 1 exhibited a 1.08 mm shift in the +*x* direction, Spot 7 shifted by 1.14 mm in the −*x* direction, Spot 23 showed a 1.13 mm shift in the +*y* direction, and Spot 19 shifted by 1.05 mm in the −*y* direction. The spot shift detection accuracy was calculated as 0.10 mm (± 0.04 mm). During testing for a presumed 1% gain or calibration error in the SPM, the film detected a gradual increase in spot error with distance from the center, reaching a maximum deviation of approximately 2 mm at the outermost spots and tapering to nearly zero at the center. It should be noted that such a scenario, causing this type of detector behavior, is considered unlikely.

## 4 DISCUSSION

This study introduces a comprehensive QA framework designed to evaluate proton gantry and couch isocentricity, spot position accuracy, and spot size consistency using the custom‐designed Octopoint phantom and Gafchromic film analysis. The framework effectively assesses end‐to‐end isocentricity through a streamlined workflow centered around the Octopoint phantom. Precise measurements of spot position accuracy and spot size are obtained through Gafchromic film analysis, ensuring the high precision necessary for radiotherapy treatments. The integration of SPM log file analysis further strengthens the system's robustness, providing a dual‐modality approach that enhances the verification and accuracy of treatment delivery. Importantly, this framework is adaptable to other institutions, advancing quality assurance processes and ensuring reliability and consistency across different clinical scenarios.

As shown in Figure 6a, differences in isocentricity are apparent between the gantries. During the survey period, Gantry Rooms 1 and 4 demonstrated better isocentricity compared to Rooms 2 and 3, with an average difference of around 0.4 mm. Although these differences are small, they may have clinical significance, especially for stereotactic body radiation therapy (SBRT), which requires precise patient positioning. Scheduling SBRT treatments on gantries with superior isocentricity could potentially improve accuracy. Figure 6b shows data acquired immediately after IGRT, reflecting the residual error between the PIAS imaging and the delivery system in a 2D beam's eye view. The larger error observed in Figure 6c compared to Figure 6b is likely due to couch runout and inaccuracies associated with virtual isocenter corrections. Since the time of data collection summarized here, we have been actively collaborating with our vendor to further improve isocentric performance. Improvements have been realized, but these gains have typically been at the expense of larger virtual isocenter corrections, which we feel is a reasonable tradeoff.

The results of the Octopoint repeatability and sensitivity tests confirm the system's precision and reliability in clinical use. In the repeatability test, the maximum standard deviation was 0.1 mm, demonstrating Octopoint's ability to consistently provide accurate measurements. This high precision is essential for maintaining treatment accuracy, particularly in protocols with tight tolerances. The sensitivity test further supports Octopoint's reliability, showing that the system effectively detects small, intentional offsets. Due to the system's geometric design and constraints over gantry and PPS rotation combinations, we observed higher sensitivity to erroneous shifts along the PPS *y*‐axis (as evidenced by more frequent warnings and failures under the same shift conditions compared to intentional shifts introduced to the PPS *x* and *y* axes). However, from a practical standpoint, hindsight over approximately one decade of clinical operation has taught us that we very rarely detect any Octopoint films exceeding our warning threshold; when we do, something has likely changed on the vendor side, or a setup error has been made during the test. We are easily able to discern between these two scenarios by repeating our measurement.

Year‐long monitoring of spot position and size deviations (Figure 7a,c) indicates that these measurements are sensitive to changes in QA procedures and hardware. In May of this reported evaluation window, we updated our baseline measurement methods following a revision of the divergence‐commissioning process. Previously, the calibration grid only included spots in the positive quadrant (“*y*‐positive” film) as shown in Figure 4. The new approach incorporated a small geometry correction for the Epson flatbed scanner (0.35% lateral and −0.17% longitudinal), which had been previously overlooked. As seen in Figure 7a,c, this update reduced the film‐based spot position error, although it does not necessarily reflect a decrease in absolute spot position error. Additionally, the spread in spot position error, indicated by the range of the box plot, decreased following the calibration update. In September of the evaluation window, synchrotron and beam‐line tuning adjustments were made to prepare for multi‐energy extraction (MEE) capability, which likely explains the increase in spot size errors observed during the September–October period for the tracked gantry.

Figure 7b,d shows the distribution of spot position errors over time as a function of spot number, corresponding to both location and energy (see Figure 4 for the pattern). The data suggests a correlation between spot position error and either distance from the center of the SPM, energy, or both. In Figure 7b, the spot position error follows a U‐shaped pattern, with spots closer to the center, particularly higher‐energy spots like #12 and #14, showing smaller errors. This pattern is expected, as the center spot (#13) is anchored at the origin in the film analysis, leading to more systematic errors for spots further from the center. In contrast, the relationship between spot size error and distance or energy is more complex, showing no clear pattern. This reflects the combined influence of energy and distance, making the correlation less apparent.

In Figure 8a, the close proximity of the combined spot size ratios to unity indicates strong agreement between SPM‐log predictions and the spot sizes measured on film. This alignment suggests that both the SPM and film analyses independently confirm the stability of spot sizes, supporting the reliability of using log‐file analysis for patient‐specific QA.^22^ The slight deviations observed are likely due to minor variations in the film measurement process or inherent uncertainties in beam delivery, but these deviations are minimal and remain within clinically acceptable limits. Comparisons of absolute spot positions from SPM and film data (not shown for brevity) further support the consistency of these results, showing no unexpected discrepancies.

Figure 8b highlights trends in the fitting parameters. Notably, there is greater variation in the focal length parameter, which is expected given its direct relationship to gantry‐specific beam tuning. The physical distance between the active area of the SPM and the isocenter plane is approximately 530 mm, aligning well with the average focal length values obtained, reinforcing confidence in the parameterization and the vendor's beam tuning process. In contrast, the MCS coefficient, which relates to a physical process, is expected to remain consistent across all gantries, assuming identical physical setups between the SPM and the range shifter. To maintain accuracy, we track the MCS coefficient as a running average. Going forward, monitoring the focal length parameter while assuming constancy of the MCS coefficient could offer valuable insights into gantry‐specific spot size changes or potential issues. Supporting this approach is the observed increase in combined spot size error, though sub‐clinically significant, following the SEE beam tuning change in September (Figure 7c). Around the same time, a notable decrease in focal length values (Figure 8b) was consistently observed across all gantries, suggesting a possible link between these changes and beam tuning adjustments. However, this remains an ongoing area of research, and further data is needed to establish a definitive relationship between the fitting parameters and spot size or position errors.

The spot position sensitivity tests confirm the system's robust ability to detect and accurately reflect intentional shifts, both constant and percentage‐based, across all positions. The consistent measurements across the field demonstrate the system's precision in managing both uniform and random positional changes. The system also effectively responds to simulated 1% gain or calibration errors, detecting increasing deviations at the periphery. These results highlight the system's reliability and its ability to handle changes across all positions, ensuring precise dose delivery in clinical settings where accuracy is critical.

Despite the promising results, several limitations of the described QA workflow must be reinforced. First, regarding our spot position and size test, the use of the range shifter as a positioning template and Gafchromic film as the detector, while convenient and practical, introduces additional sources of error and complexity into the analysis—particularly due to the lack of a true ground‐truth for determining the beam origin. Thus, our calculation of “spot position” accuracy relies on the isocentricity of the center spot. So as to indirectly recover absolute position information, our standard practice is to perform this spot position QA immediately after Octopoint QA. Therefore, we interpret our spot position QA test as being sensitive to changes from a defined baseline, or that is, being sensitive to spot position changes relative to the center spot's position. Second, relating to our theoretical parameterization of spot growth as a means to monitor our vendor's beam tuning, the relationship between focal length, the MCS fit coefficient, and the beam delivery system is not fully explored. For example, the assumption of minimal energy dependence for the focal length F(β) is tenuous and may explain the slightly poorer combined spot size ratio observed at higher energies. Incorporating an energy‐dependent F(β) fit could potentially provide a more accurate indicator of system stability. Further trend monitoring and analysis are required to better understand how these parameters can be effectively used to monitor hardware stability. Third, our described workflow is limited to evaluating isocentricity, spot size, and spot position. Further optimization and the inclusion of other recommended monthly tests would enhance its effectiveness. For example, adding one or two wedges or a semicircle to the Octopoint phantom could enable energy constancy checks at different gantry angles within the same setup,^23^ thereby streamlining the monthly QA workflow. Fourth, the current analysis process relies heavily on MATLAB‐based code, which involves extensive manual input and is time‐consuming. Streamlining and automating this process would improve efficiency and reduce the potential for human error. Lastly, the framework currently relies on a custom phantom and in‐house developed software, which limits its adaptability to other clinics at this stage. Future efforts will focus on improving its generalizability by making the majority of the Octopoint phantom 3D‐printable and creating a Docker package to encapsulate the MATLAB code, allowing it to be easily deployed across different operating environments.

## 5 CONCLUSIONS

This study demonstrates the effectiveness of a comprehensive QA framework designed to ensure high precision in proton therapy delivery. By integrating the custom Octopoint phantom, Gafchromic film analysis, and SPM log file data, the framework provides reliable measurements of isocentricity, spot position accuracy, and spot size consistency under various conditions. The approach has proven its reliability and clinical applicability, contributing to improved accuracy and safety in proton therapy treatments.

## AUTHOR CONTRIBUTION

*Writing—original draft, Writing—review & editing, Methodology, Formal analysis: Xueyan Tang. Conceptualization, Methodology, Writing—review & editing, Data Curation: Chris J. Beltran. Conceptualization, Methodology, Writing—review & editing, Data Curation: Keith M. Furutani. Conceptualization, Methodology, Writing—review & editing, Data Curation: Graham S. Gilson. Data Curation, Writing—review & editing: Jon Gustafson. Conceptualization, Methodology, Writing—review & editing, Data Curation: Michael G. Herman. Conceptualization, Methodology, Data Curation, Writing—review & editing, Data Curation: Shima Ito. Conceptualization, Methodology, Writing—review & editing, Data Curation: Jed E. Johnson. Conceptualization, Methodology, Writing—review & editing, Data Curation: Jon J. Kruse. Data Curation, Writing—review & editing, Data Curation: Kenneth M. Long. Conceptualization, Methodology, Writing—review & editing, Data Curation: Daniel W. Mundy. Conceptualization, Methodology, Writing—review & editing, Data Curation: Nicholas B. Remmes. Conceptualization, Methodology, Data Curation, Writing—review & editing: Ali M. Tasson. Conceptualization, Methodology, Data Curation, Writing—review & editing: Thomas J. Whitaker. Methodology, Writing—review & editing: Witold Matysiak. Conceptualization, Methodology, Formal analysis, Writing—original draft, Writing—review & editing, Data Curation, Supervision, Project administration: Erik J. Tryggestad*.

## CONFLICT OF INTEREST STATEMENT

The authors have no conflicts to disclose.

## Data Availability

The data that support the findings of this study are available from the corresponding author upon reasonable request.
